# JOSEPH T. FREEMAN AWARD AND EXCELLENCE IN REHABILITATION OF AGING PERSONS AWARD PRESENTATIONS AND LECTURES

**DOI:** 10.1093/geroni/igac059.836

**Published:** 2022-12-20

**Authors:** Cynthia Brown

## Abstract

The Joseph T. Freeman Award lecture will feature an address by the 2022 Freeman Award recipient Neil M. Resnick, MD, FGSA, of the University of Pittsburgh. The Joseph T. Freeman Award is a lectureship in geriatrics awarded to a prominent clinician in the field of aging, both in research and practice. The award was established in 1977 through a bequest from a patient's estate as a tribute to Dr. Joseph T. Freeman. The Excellence in Rehabilitation of Aging Persons Award lecture will feature an address by the 2022 Excellence in Rehabilitation Award recipient Patricia C. Heyn, PhD, FGSA, FACRM, of Marymount University. The Excellence in Rehabilitation of Aging Persons Award is designed to acknowledge outstanding contributions in the field of the rehabilitation of aging individuals.

